# Preventive effect of memory foam position pads on intraoperative pressure-induced skin injuries in gynecological malignancy patients undergoing lithotomy positioning

**DOI:** 10.1097/MD.0000000000043544

**Published:** 2025-08-01

**Authors:** Miao Song, Jianping Feng, Jincui Wei, Lijie Yan, Yangyang Guo, Qian Zhang

**Affiliations:** a Department of Anesthesiology and Perioperative Medicine, The First Affiliated Hospital with Nanjing Medical University, Nanjing, China.

**Keywords:** compressed skin, lithotomy, memory sponge pad, stress injury

## Abstract

This study explores the effect of memory sponge body position pad on pressure injury of compressed skin in patients undergoing lithotomy. From October 2021 to March 2022, 94 adult patients with gynecological malignancies who underwent elective general anesthesia surgery in our hospital and were in the lithotomy position during the operation were selected as the research objects. According to the different kinds of nursing, they were divided into 47 cases in the control group and 47 cases in the experimental group. The control group used silicone gel pads, and the experimental group used memory sponge pads. The incidence of pressure injury (PI), postoperative comfort contact area and average pressure of pressure part, and the surface microenvironment of the pressure part were compared between the 2 groups. Compared with the control group (17.02%), the incidence of PI in the observation group (2.13%) was significantly lower (*P* < .05); Compared with the control group (12.77%, 17.02%, 21.28%), the incidence of limb numbness (0.00%), waist muscle pain (4.26%) and neck and shoulder pain (6.38%) in the observation group were significantly lower (*P* < .05); Compared with the control group, the contact area of the compressed part in the observation group increased significantly and the average pressure decreased significantly after anesthesia, after positioning, during and after operation (*P* < .05); Compared with the pressure for 1 hour, the humidity and temperature of the feet, buttocks, and shoulders of the 2 groups were significantly higher after 2 hours of pressure, while the humidity and temperature of the feet, buttocks, and shoulders were significantly lower in the observation group compared to the control group (*P* < .05). The memory sponge position pad can prevent the occurrence of PI on the compressed skin of patients undergoing lithotomy and can also improve the postoperative comfort of patients and the surface microenvironment of the compressed part, which has high clinical reference value.

## 1. Introduction

Pressure injury (PI), also known as pressure ulcer, pressure sore and bedsore, is a localized injury caused by clipping force, friction force, and vertical pressure acting on the skin at the femoral tuberosity or (and) potential subcutaneous tissue singly or in combination, and is prone to occur in the ankle joint, heel, elbow, underside of knee, occipital, hip, and coccyx.^[1,2]^ With the rapid development of surgical technology, complex and long-time major surgery has been widely used in clinical practice. The suitable position for surgery has a positive significance for the smooth development of the surgery. The lithotomy position is one of the commonly used positions.^[3]^ However, the lithotomy position is a forced position. The long-time placement of this position can make the local skin in a long-term stress state, which is not conducive to blood circulation, and then causes the PI of the knees and sacrococcygeal region, including skin tissue damage, induration, blisters and redness. This not only increases the pain perception and economic burden of patients, but even increases the risk of death, requiring high clinical attention.^[4]^ Although in-depth research was conducted in the aspects of improving the surgical position and selecting the surgical pad in the early clinical stage, the incidence of PI remains high, affecting the patients’ feelings of seeking medical treatment and postoperative rehabilitation.^[5]^ PI occurs based on tissue tolerance and pressure. Tissue tolerance is related to the microenvironment and its own factors, while pressure is related to the time and degree of compression.^[6]^ In recent years, some scholars have shown that changing the surrounding or surface microenvironment of the skin has a positive clinical significance in reducing the occurrence of PI.^[7]^ Memory foam pad has good anti-pressure and elasticity and can reduce the damage through suction and pressure buffering.^[8]^ However, research on its effect on PI is still rare, and it is worthy of further study. Therefore, this study’s aim was mainly to explore the effect of memory sponge pad on the PI of compressed skin in patients undergoing lithotomy position, aiming to provide more references for improving the clinical treatment efficiency and alleviating the prognosis.

## 2. Material and methods

### 2.1. General material

This study was approved by the Ethics Committee of the First Affiliated Hospital with Nanjing Medical University. A total of 94 adult patients who underwent elective gynecological malignant tumor surgery under general anesthesia in our hospital from October 2021 to March 2022 and whose intraoperative position was lithotomy position were retrospectively selected as the research subjects. They were divided into the control group (n = 47) and the experimental group (n = 47) according to the different kinds of nursing, with the silicone gel pad in the control group and the memory sponge pad in the experimental group. The differences in age, weight, and pathological type (noninvasive ductal carcinoma, common type of ovarian carcinoma, surgical duration, anesthesia type, and common type of endometrial carcinoma) between the 2 groups were not statistically significant (*P* > .05), with comparability, as shown in Table 1.

**Table 1:** Comparison of 2 groups of general data.

General material		Control group (n = 47)	Observation group (n = 47)	*t*/*χ*^2^ value	*P* value
Average age (years)		42.33 ± 4.21	41.78 ± 5.47	0.372	.708
Average weight (kg)		62.89 ± 4.49	63.37 ± 5.73	0.459	.639
Pathological type [n (%)]	Ovarian cancer	18 (38.30)	16 (34.04)	0.473	.492
	Carcinoma of endometrium	29 (61.70)	31 (65.96)		
Surgical duration (hours)		2.5 ± 0.7	2.6 ± 0.8	0.465	.643
Anesthesia type [n (%)]					
General anesthesia		40 (85.11%)	42 (89.36%)	0.218	.641
Regional anesthesia		7 (14.89%)	5 (10.64%)		

### 2.2. Criteria of inclusion and exclusion

Inclusion criteria: ① patients who met the surgical indications of gynecological pelvic malignant tumors; ② Aged 18 years old, with normal speech and hearing ability, informed consent and voluntary participation; ③ Patients who were under general anesthesia and classified as grades I–II by the American Society of Anesthesiologists;^[9]^ ④ Patients whose operation position was in the lithotomy position; ⑤ The operation time was 3 hours (from skin incision to the end of the operation); ⑥ The postoperative hospital stay ≥ 3 days; and ⑦ The patient or his/her family member signs an informed consent form.

Exclusion criteria: ① Patients with severe joint dysfunction; ② Patients with acute and chronic skin diseases or PI in skin and mucosa; ③ Patients with postoperative consciousness disorder or admitted to ICU; and ④ Patients whose operation plans were changed or who withdrew from the study due to other reasons.

### 2.3. Research methods

The near-infrared tissue oxygen parameter detection instrument was used to monitor the tissue oxygen changes in the compression parts of patients during the operation, and the tissue oxygen value was directly read out by the monitoring machine after the operation. The Tactilus pressure sensing system was used for continuous monitoring and real-time recording of the average pressure and the peak pressure of the compressed parts and the total contact area of the patient and the operating bed in different time periods during the operation. Before patients entered the operation room, the operation bed was prepared, and posture pads were placed on the operation bed. The control group placed silicone gel pads at the sacrococcygeal region, and the experimental group placed memory sponge pads at the sacrococcygeal region, with the specification of 80 × 50 × 8 cm. The memory sponge pads were divided into 2 layers, with the upper layer being memory sponge, and the lower layer being common sponge, both of which were 4cm in thickness, and the outer layer being removable cotton cloth. The pressure test pad was paved on the surgical position pad and connected with the computer Tactilus system to debug and detect the pressure measurement system. The patient was then guided to place the modified lithotomy position so that the thigh was 130° with the longitudinal axis of the body and 90° with the longitudinal axis of the calf.

### 2.4. Research methods

All assessments were performed by a team of trained evaluators using a dual-assessment method to ensure consistency and reduce inter-rater variability. The evaluators underwent standardized training to follow the established criteria for assessment.

① PI occurrence: PI was defined and divided into stages by unified trained team members according to the National Pressure ULCER Advisory Panel.^[10]^ After the operation, the skin redness (3 seconds fading after finger pressure at the pressure part of the skin) and the skin PI location, degree and area were observed. PI was divided into 4 stages, stage I refers to red and hot skin pain, but no damage to deep tissues. Stage II refers to the existence of blisters, lumps and induration in local skin tissue, but no damage to bones and muscles. Stage III refers to the ulcer formed by superficial tissue, manifested as local skin defect, with pus and abscess in the skin and subcutaneous tissue; Stage IV deep ulcer occurs, which includes not only ulcer and necrosis of skin and subcutaneous tissue, but also necrosis and destruction of bone and muscle. ② Postoperative comfort level: 12 to 24 hours after operation, when the patient had clear consciousness, normal perception function and clear speech expression ability, he/she was asked whether he/she suffered from limb numbness, psoas pain, neck and shoulder pain and other uncomfortable symptoms. ③ Contact area and average pressure of the compressed parts: The Tactilus pressure sensing system was used to record the contact area and average pressure of the skin at the compressed part and the operating bed in the 2 groups of patients after anesthesia, posture placement, during and after surgery. ④ Surface microenvironment of the compression part: The surface humidity and temperature of the compressed skin in the foot, hip and shoulder of the patients in the 2 groups were measured with an electron gun measuring instrument and a wired body temperature measuring instrument at 1 and 2 hours during the operation.

### 2.5. Statistical methods

Statistical analysis was performed using SPSS 17.0. The measurement data were expressed as mean standard ± deviation $\left(\bar{x}\pm s\right)$ and tested using *t*; Enumeration data were expressed as example (N) or percentage (%) and *χ*^2^ test was conducted. *P* < .05 indicated that the difference had statistical significance.

The sample size was calculated based on the primary outcome of PI incidence, with an expected effect size of 0.5, a power of 80%, and a significance level of 0.05 (α = 0.05). The calculation indicated that a minimum of 47 participants per group would provide sufficient power to detect a statistically significant difference in PI incidence between the control and experimental groups. This sample size also accounted for a potential dropout rate of up to 10%.

## 3. Results

### 3.1. Comparison of the occurrence of 2 groups of PI

The incidence of PI in the observation group was significantly lower than that in the control group and the difference was statistically significant (*P* < .05), as shown in Table 2.

**Table 2:** Comparison of the occurrence of 2 groups of PI [n (%)].

PI occurrence	Control group (n = 47)	Observation group (n = 47)	*χ*^2^ value	*P* value
Stage I	0 (0.00)	0 (0.00)	–	–
Stage II	6 (12.77)	1 (2.13)	–	–
Stage III	2 (4.26)	0 (0.00)	–	–
Stage IV	0 (0.00)	0 (0.00)	–	–
Total occurrence	8 (17.02)	1 (2.13)	4.453	.034

PI = pressure injury.

### 3.2. Comparison of postoperative comfort between the 2 groups

The incidence of limb numbness, psoas pain, neck and shoulder pain in the observation group was significantly lower than that in the control group, and the difference was statistically significant (*P* < .05), as shown in Table 3.

**Table 3:** Comparison of postoperative comfort between the 2 groups [n (%)].

Discomfort symptoms	Control group (n = 47)	Observation group (n = 47)	*χ*^2^ value	*P* value
Limb numbness	6 (12.77)	0 (0.00)	4.453	.034
PsoS pain	8 (17.02)	2 (4.26)	4.031	.044
Neck and shoulder pain	10 (21.28)	3 (6.38)	4.376	.035

### 3.3. Comparison of contact area and average pressure of 2 groups of pressure parts

The contact area at the compression part in the observation group after anesthesia, posture placement, operation and completion of operation was significantly larger than that in the control group, and the average pressure was significantly lower than that in the control group. The differences were statistically significant (*P* < .05), as shown in Table 4.

**Table 4:** Comparison of contact area and average pressure of 2 groups of pressure parts (±*s*).

Item		Control group (n = 47)	Observation group (n = 47)
Contact area (cm^2^)	After anesthesia	2167.06 ± 430.26	2485.32 ± 384.18*
	After posture placement	2107.81 ± 366.32	2374.32 ± 438.01*
	During operation	2262.00 ± 363.00	2587.47 ± 430.79*
	After operation	2512.93 ± 369.79	2825.00 ± 461.01*
Average pressure (mm Hg)	After anesthesia	25.66 ± 6.69	19.30 ± 3.22*
	After posture placement	27.93 ± 6.41	23.17 ± 3.78*
	During operation	28.78 ± 6.79	24.53 ± 4.26*
	After operation	30.21 ± 6.96	26.10 ± 4.37*

* There was a significant difference between groups at the same time point (*P* < .05).

### 3.4. Comparison of surface microenvironment of 2 groups of pressure parts

There was no significant difference in the humidity and temperature of foot, hip and shoulder between the 2 groups 1h after compression (*P* > .05). Humidity and temperature of foot, hip and shoulder in the 2 groups were significantly increased after 2 hours of compression, while those in the observation group were significantly lower than those in the control group (both *P* < .05), as shown in Table 5.

**Table 5:** Comparison of surface microenvironment of 2 groups of pressure parts.

Item		Control group (n = 47)		Observation group (n = 47)	
		After 1 h compression	After 2 h compression	After 1 h compression	After 2 h compression
Humidity (%)	Foot	26.18 ± 1.07	31.11 ± 1.30*	25.77 ± 1.03	29.33 ± 1.44*^,^†
	Hip	34.73 ± 1.46	42.57 ± 1.99*	35.18 ± 1.57	37.53 ± 1.76*^,^†
	Shoulder	32.58 ± 1.27	36.54 ± 1.41*	33.04 ± 1.11	34.67 ± 2.28*^,^†
Temperature (°C)	Foot	31.41 ± 0.97	32.54 ± 1.09*	31.53 ± 0.91	32.06 ± 0.98*^,^†
	Hip	34.87 ± 0.34	35.62 ± 0.38*	34.48 ± 0.26	35.04 ± 0.31*^,^†
	Shoulder	34.69 ± 0.31	35.67 ± 0.26*	34.31 ± 0.29	35.03 ± 0.22*^,^†

* *P* < .05 indicates the comparison within the same group at different time points.

† *P* < .05 indicates the comparison between groups after 2 h of compression.

## 4. Discussion

PI refers to the acting force exerted on the contact surface of 2 objects, which is mostly local damage of cartilage and skin tissue, presenting as open or complete skin ulcer with pain.^[11]^ Shear force, friction force, humidity, and pressure were that main factor contributing to PI, while the direct effect of long-term vertical pressure on local tissue during the operation was the most important factor contributing to PI.^[12]^ The posture pad is one of the common appliances for clinical prevention of PI. The traditional sponge pad is formed by filling sponges with different densities and hardness, and the outer layer is coated with synthetic leather, so the permeability is poor. If the posture pad is used for a long time, there will be insufficient supporting force and local depression, which may still cause PI. Therefore, other more efficient prevention methods need to be found.^[13]^ The memory foam pad uses polyurethane foam with slow rebound property, and the special foaming process promotes the intercommunication between the vesicles, which can ensure the required hygroscopicity and breathability of the body.^[14]^ However, there are few studies exploring the effect of memory foam pads on the PI of compressed skin in patients undergoing lithotomy position. The results of this study showed that the memory foam pad could reduce the occurrence of PI of compressed skin in patients undergoing lithotomy position, improve the postoperative comfort, increase the contact area of the compressed part, reduce the average pressure, and improve the microenvironment. The reasons are now analyzed as follows.

The results of Kim et al^[15]^ showed that alternating pressurized air mattresses had certain clinical significance for the prevention of PI. In this study, the PI incidence rate in the observation group was significantly reduced compared to the control group (2.13% vs 17.02%), which aligns with Kim findings. The reduced incidence of PI in the observation group can be attributed to the memory foam pad’s ability to distribute pressure more evenly across the body. Unlike traditional materials, the memory foam’s stress-relaxation characteristics allow it to better conform to the body’s contours, effectively minimizing localized pressure on critical areas. This material property leads to improved pressure distribution, reducing the risk of tissue ischemia and hypoxia, which are key contributors to PI formation. The viscoelastic nature of memory foam provides enhanced cushioning and pressure relief by absorbing and redistributing the force applied to the skin. These mechanisms are particularly important during prolonged surgeries, such as those in the lithotomy position, where sustained pressure on specific body areas is common. Thus, the memory foam pad’s unique stress-relaxation ability plays a critical role in preventing PI by ensuring more even pressure distribution, enhancing tissue perfusion, and reducing the likelihood of injury.

Additionally, this study revealed that the contact area at the compression part in the observation group after anesthesia, posture placement, operation, and completion of the operation was significantly larger than that in the control group. The average pressure was also significantly lower in the observation group compared to the control group.

The humidity and temperature of the feet, buttocks, and shoulders in both groups increased after 2 hours of compression. However, compared to the control group, the humidity and temperature in these areas were lower in the observation group. In summary, the results suggest that the memory foam pad may be used to prevent PI on the compressed skin of patients undergoing lithotomy position surgery by increasing the contact area, reducing average pressure, and controlling the temperature and humidity.

The rationale for these findings is as follows: long-term compression of local tissues can lead to ischemia and hypoxia, which can result in PI. Increasing the contact area of the compressed region and controlling the intensity of the pressure can help prevent PI.^[16]^ In the lithotomy position, patients must maintain this position throughout the surgery after anesthesia, which can increase the pressure on certain areas. The memory foam pad can facilitate rapid pressure distribution, reducing the duration of high-intensity pressure and minimizing damage to the compressed regions.

Average pressure can reflect the effect of pressure on the body. The lower the average pressure, the more the position pad aligns with the physiological structure of the body. A larger contact area between the body and the pad enhances pressure dispersion and reduces average pressure. Therefore, the pressure and contact area in each part of the lithotomy position demonstrated a typical positive correlation with the duration of the surgery. As anesthesia takes effect, patients enter a deeper state, accompanied by muscle relaxation and increased depth of anesthesia, which may affect the stability of the position. The memory foam pad helps promote local pressure relaxation and enhances pressure protection on the compressed areas.^[17–19]^

Microenvironment refers to the humidity and temperature of the tissue or skin surface. Increased humidity and temperature are key factors in the development of PI. The friction coefficient between the skin and support surface is influenced by the type of contact surface and moisture permeability. Excessive humidity increases the friction coefficient, leading to greater shear force, which raises the risk of PI.^[20]^ An increase of 1°C in body temperature can result in a 10% increase in oxygen utilization and metabolic rate, which places tissues in a state of hypoxia, ischemia, and nutrient deficiency, increasing PI susceptibility.^[20]^ The memory foam pad’s cellular structure ensures the required hygroscopicity and air permeability for the skin. Additionally, the molecular rotation property of the foam, along with intermolecular Van der Waals forces, helps the material adapt to the shape of the compression surface by stress relaxation, thereby absorbing impact forces and reducing vibrations.^[21]^

The research of Xue et al^[22]^ demonstrated that the use of specialized posture pads after percutaneous nephrolithotomy significantly improved patient comfort and reduced postoperative complications. In our study, the incidence of limb numbness, psoas pain, and neck and shoulder pain in the observation group was significantly lower than in the control group, which aligns with Xue findings. This suggests that the memory foam pad can improve the postoperative comfort of patients undergoing lithotomy position surgery.

The reasons for this improvement are as follows: To fully expose the surgical field during lithotomy position surgery, traction and adjustments are often made based on the operation area, which may compress the related nerves. Prolonged compression can lead to symptoms such as limb numbness and psoas pain. The memory foam pad increases the contact area of the compressed region, alleviating nerve traction pressure and improving patient comfort. Additionally, the pad conforms to the body’s curves, providing support to the waist, thus effectively reducing waist pain.^[23,24]^

This study has several limitations. First, the sample size of 94 patients may be too small, which could limit the generalizability of the results. Larger studies are needed to confirm these findings and strengthen the evidence. Second, the lack of long-term follow-up means that the sustained effects of memory foam pads on pressure injury prevention remain unknown. A follow-up study would help assess whether the benefits observed in the short term extend over time and whether chronic or delayed pressure injuries could occur. Additionally, the study did not explore other long-term outcomes, such as the impact on patients’ quality of life, mobility, or additional treatments needed due to pressure injuries. Another limitation concerns the uniformity of PI assessment. Although the study followed NPUAP criteria for PI staging, variability in interpretation and application by different clinicians may have occurred. To reduce bias, PI evaluations were conducted by a trained team using a dual-assessment method. However, as a retrospective study, subjective factors, such as clinician experience, could still influence results. Future studies should incorporate standardized training and calibration to minimize inter-rater variability and improve assessment consistency. Moreover, due to the retrospective nature of this study, potential confounders such as patient comorbidities, nutritional status, and mobility levels could not be fully controlled. These factors may influence the recovery process and pressure injury prevention. Future studies should address these factors to improve the accuracy of results. Lastly, the study was conducted at a single center, which may introduce biases related to local practices and patient demographics. Future multicenter trials with larger sample sizes and longer follow-up are necessary to address these issues and further validate the effectiveness of memory foam pads in different patient populations.

In summary, the application of the memory foam pad to patients undergoing lithotomy position not only prevents PI from occurring on the compressed skin, but also improves the postoperative comfort of patients, increases the contact area of the compressed part, reduces the average pressure, and improves the microenvironment, which is worthy of clinical reference.

## Acknowledgments

This work was supported by Jiangsu Provincial People’s Hospital “Clinical Capability Enhancement Project” Nursing Project (No. 2021.20).

## Author contributions

**Conceptualization:** Miao Song, Jianping Feng, Jincui Wei, Lijie Yan, Yangyang Guo.

**Data curation:** Miao Song, Jianping Feng, Lijie Yan, Qian Zhang.

**Formal analysis:** Miao Song, Jianping Feng, Lijie Yan, Yangyang Guo, Qian Zhang.

**Funding acquisition:** Yangyang Guo.

**Investigation:** Miao Song, Lijie Yan, Qian Zhang.

**Methodology:** Miao Song, Jincui Wei, Lijie Yan, Qian Zhang.

**Software:** Jianping Feng.

**Supervision:** Jianping Feng, Jincui Wei, Yangyang Guo.

**Visualization:** Yangyang Guo.

**Writing – original draft:** Miao Song, Qian Zhang.

**Writing – review & editing:** Miao Song, Qian Zhang.
